# Report of M. Dupuytren, Anatomical Professor in the School of Medicine at Paris, in the Name of a Commission, Composed of Messrs. Cuvier, Leroy, Baudelocque, and Jadelot; Respecting a Fœtus, Found in the Body of a Youth of Fourteen Years Old. Extracted from the Bulletin of the Society

**Published:** 1805-08-01

**Authors:** 


### *Report of M.* Dupuytren, *Anatomical Professor in the School of Medicine at Paris, in the Name of a Commission, composed of Messrs.* Cuvier, Leroy, Baudelocque, and Jadelot; *respecting a* Fœtus, *found in the Body of a Youth of Fourteen Years old. Extracted from the Bulletin of the Society.*

AMIDEE Bissieu, in whose body the fœtus was found, had complained from a very early period of his existence, of a pain in the left side, which soon assumed the form of a tumor. These symptoms continued, without producing any material injury to the health or faculties of the child, until he had attained his 13th year, when he was suddenly seized with fever. From that period the tumor increased in size, and became very painful; after a few days, his stools were observed to be fœtid and puriform. At the termination of three months from this attack, he evidently laboured under a species of pulmonary consumption. Shortly afterwards, afterwards, he voided by stool a ball of hair; and at the end of six weeks, he sunk under his complaints.

On the body being opened by Messrs. Guérin, and Bertin, there were discovered in a sac, attached to the arch of the colon, and communicating with it, some balls of hair, and an organized mass, somewhat resembling a human fœtus. The connection between the habitual indisposition of young Bissieu and his disease is obvious, as well as that betwixt the disease and the appearances on dissection. The first point being ascertained by authentic documents, it became of the greatest importance to determine the position of the organized mass, and the situation in which it had been evolved.

From a careful inspection of the parts sent to the society by M. Blanche, surgeon, at Rouen, it clearly appears to have been contained in a cyst, situated in the transverse mesocolon, near the colon, and without the canal of the primæ viæ. The cyst indeed communicated with the intestine; but this communication was recent, and appeared to be in a great measure accidental; as the remains of the separation between the two cavities were still evident.

The true position of the organized mass being determined, it was next desirable to ascertain its nature. In its form, we discovered many points of resemblance with the human fœtus; but we likewise observed various particular dispositions, some of which appeared to belong to a defective conformation, and others to the derangements successively occasioned by time, and by its retention in the mesocolon.

It is evident, however, that if this production possessed a system of organs independent of those of the body to which it was attached, it must necessarily be regarded as an individual being; but that, on the contrary, if nothing could be discovered but a continuous organization, it ought to be referred, whatever might be its external form, to the class of tumors which originate in every part of organized bodies. The dissection of this mass, performed with the greatest care and accuracy, discovered traces of some of the organs of sense: viz. a brain, a spinal marrow, and large nerves, muscles degenerated into a kind of fibrous matter; a skeleton composed of a spine, a head, a pelvis, and the rudiments of the limbs; lastly, in a very short umbilical cord, inserted into the mesocolon without the cavity of the intestine, there were found an artery and a vein ramified at their respective extremities, both towards the fœtus, and towards the individual to whom it was attached.

tached. The existence of the preceding organs clearly demonstrates the individuality of this organized mass, although it was destitute of those of digestion, of respiration, of urine, and of generation; but the absence of these organs, necessary to life, ought only to make us regard it as one of those monstrous fœtuses destined to perish at the moment of birth.

The existence of an organized mass in the mesocolon being ascertained, and its analogy with the human fœtus determined, it still remained for us to inquire how long it had existed, and in what manner it had been evolved, and nourished in the body of another individual.

This fœtus being found without the alimentary canal, it could not be introduced into the body of the young Bissieu after birth; a circumstance which overturns a great many of the hypotheses, by which it has been proposed to explain this phænomenon. The sex of Bissieu, which was carefully ascertained by Messrs. Delzeuse and Bronard, by order of the prefect of the Eure, precludes all idea of fecundation; nor could the youth be self-impregnated, since he was provided with male organs alone, and did not exhibit the smallest trace of those of the other sex.

The facts which form the basis of this report, lead naturally to very different conjectures. The indisposition to which Bissieu had been subject from childhood, the nature of the symptoms of this indisposition, as also those of the disease which immediately suceeeded it, as well as the appearances discovered on dissection, are so closely allied, that we are forced to acknowledge there must exist between them a necessary dependance, and to conclude that this unfortunate young man had brought with him into the world the cause of the disease which cut him off at the end of fourteen years.

Many other facts concur to prove, that the fœtus must have existed for a long time in the body of the young Bissieu; such as the size of the teeth, the fibrous degeneration of the muscles, the induration of the brain, the abrasion of different portions of skin, the carious state of several bones, the anchylosis of others, the ossification of the cyst, &c. But admitting that the existence of this fœtus was coeval with that of the individual to whom it was attached, it will still be difficult to explain why it should have been situated in the transverse mesocolon.

The facts detailed in this report are unquestionably the most important part of it, and are, to a certain extent, independent of any explanation which we may venture to to propose. It necessarily however became an object with your commission, to endeavour to discover in the facts themselves, an explanation of the phænomenon. We frequently meet with twins, connected by the back, belly, head, or other parts of the body. A certain degree of compression, exerted by the organs of the mother, either at the time, or immediately after conception, may probably occasion these, or similar monstrosities. In other cases, not less frequent, twins are found so identified, that several organs wanting in each, are re-placed by others common to both. In the former case, the monstrosity is owing to a cause purely mechanical, and in the latter, to a radical defect in the organization of the germs. In order to explain the phænomenon, which is the subject of the present report, we must necessarily have recourse to one or other of these causes. Thus, in the case of Bissieu, either one or two separate germs must have penetrated the other in consequence of some mechanical action; or from an original disposition, for which it would be as difficult to account, as for many other circumstances connected with generation; they must have always existed in the same relative situation in which we found them. One of these explanations being admitted, the existence of a fœtus in the abdomen of an individual is no longer a matter of great surprise; and the sex of that being, who for so long a time performed the functions of mother, becomes nearly a matter of indifference. This fœtus may be compared to the fruits of extra uterine conceptions. In fact, to whatever part of the abdomen the fecundated germs are attached, their mode of nutrition is the same; in every situation they absorb nourishment by means of their own vessels; they increase in size until the term fixed by nature for their expulsion; if this be not effected at the appointed time, they putrefy, are converted into fat, shrivel, ossify, or else, by the irritation they produce in the neighbouring parts, an abscess is formed, through which they may be expelled. In the present instance, this had in some degree taken place; the sides of the cyst inclosing the fœtus, had inflamed, as in other cases of extra uterine gestation; the inflammation had extended to the intestine; the partition which separated the two cavities had been destroyed; a communication had been formed between the cyst and the colon; pus and hair had been passed by stool; and a complicated phthisis had finally terminated in the death of the patient.

Had the fœtus been placed nearer the surface, most probably bably the cyst would not have opened into the intestine, and this phænomenon, without any change in its nature, would have appeared less extraordinary.

This fœtus must have received nourishment during the whole life of the individual, who may be regarded in the light of its brother; the preservation of its body from putrefaction, and the circulation of blood through its organs, leave no room for hesitation on this head. The want of the organs of digestion, of respiration, generation, &c. afford no objection to the vitality of the fœtus, since these organs, simply nourished in ordinary fœtuses, do not exert their functions till after birth. Farther, the life of this fœtus must necessarily have consisted of a small number of functions, on account of its particular structure; the organs of circulation alone performed all that was necessary to the vitality of the other parts. They received and returned the blood from the mesocolon to the fœtus, and from the fœtus to the mesocolon. H.

